# Genome-Wide Identification and Expression Profiling of the *Aux*/*IAA* Gene Family in Eggplant (*Solanum melongena* L.) Reveals Its Roles in Abiotic Stress and Auxin Responses

**DOI:** 10.3390/ijms27010350

**Published:** 2025-12-29

**Authors:** Yanyu Lin, Yutong Li, Yimeng Wang, Hayman Soe, Xuansong Yang, Wenjing Li, Hui Li, Zhixuan Zhang, Peilin Yu, Weiren Wu, Xiaofang Xie, Yan Zheng

**Affiliations:** 1 College of Life Sciences, Fujian Agriculture and Forestry University, Fuzhou 350002, China; 2 Fujian Provincial Key Laboratory of Crop Breeding by Design, Fujian Agriculture and Forestry University, Fuzhou 350002, China; 3 Key Laboratory of Genetics, Breeding and Multiple Utilization of Crops, Ministry of Education, Fujian Agriculture and Forestry University, Fuzhou 350002, China

**Keywords:** genome-wide identification, expression profiling, *Aux*/*IAA* gene family, abiotic stress, auxin, eggplant

## Abstract

The auxin/indole-3-acetic acid (*Aux*/*IAA*) gene family encodes central regulators of plant development and stress adaptation. Eggplant (*Solanum melongena*), an economically important vegetable crop, is highly susceptible to abiotic stresses, yet its *Aux*/*IAA* family remains uncharacterized. This study aimed to systematically characterize the *Aux*/*IAA* gene family in eggplant and to explore its potential roles in development and abiotic stress responses using a genome-wide approach. Here, *35 SmIAA* genes were identified through comprehensive bioinformatic analyses, including phylogenetic classification, synteny analysis, protein–protein interaction prediction, and qRT-PCR validation. Phylogenetic analysis classified these genes into Clades A and B, encompassing nine subgroups, with subgroup B4 showing lineage-specific expansion and encoding non-canonical *Aux*/*IAA* proteins. Expression profiling revealed that *SmIAA18* and *SmIAA33* were strongly responsive to salt stress, whereas *SmIAA1*/*2*/*8* were preferentially induced by drought stress. Furthermore, *SmIAA8* and *SmIAA33* exhibited contrasting responses to IAA treatment, characterized by delayed induction and rapid repression, respectively. This study presents the first genome-wide analysis of the *Aux*/*IAA* family in eggplant, elucidating its roles in development and stress adaptation, and provides valuable genetic resources for the molecular breeding of stress-tolerant varieties.

## 1. Introduction

Eggplant (*Solanum melongena* L.) is an economically important vegetable crop cultivated worldwide. According to FAOSTAT data, the global harvested area of eggplant reached approximately 1.92 million hectares in 2023, with an average yield of 31.6 t·ha^−1^ [1]. Despite its importance, eggplant production is highly susceptible to abiotic stresses such as drought and salinity, which severely impair growth, fruit development, and yield stability [2,3]. Consequently, improving stress tolerance in eggplant has become a major focus of breeding programs.

Plant responses to abiotic stresses are controlled by complex regulatory networks involving physiological, biochemical, and molecular processes [4]. Among these, plant hormones are key players in mediating how plants perceive and respond to environmental signals [5]. These hormones enable plants to integrate stress signals with their growth and developmental pathways [6,7]. Auxin, one of the most important plant hormones, regulates a wide range of developmental processes, including cell elongation, division, differentiation, and organogenesis [8,9,10,11,12,13,14,15,16,17,18,19]. In addition to these fundamental roles in development, auxin signaling is crucial for plant adaptation to abiotic stress. It helps plants perceive and respond to environmental stress signals, such as those induced by drought and salinity, thereby contributing to stress tolerance [20,21,22]. The core of auxin signaling is mediated by the TIR1/AFB–Aux/IAA–ARF pathway, where Aux/IAA proteins act as transcriptional repressors and early-response elements in regulating auxin-responsive genes [23,24]. At low auxin concentrations, Aux/IAA proteins interact with ARFs to repress downstream genes, whereas increased auxin levels lead to the degradation of Aux/IAA proteins, enabling ARFs to activate stress-responsive genes [9,25,26,27,28,29].

As a core regulatory factor in plant growth and development, the *Aux*/*IAA* gene family has been extensively studied since its crucial role in auxin signal transduction was revealed [6,7]. Recent studies have focused on their functional diversity, particularly in regulating root development, lateral root formation, and stress responses. For instance, in *Arabidopsis thaliana*, multiple *AtIAA* genes interact with the ARF family to regulate root growth and gravitropic responses [30]. Similarly, in *Oryza sativa* (rice), *OsIAA23* and *OsIAA19* play key roles in grain development, as demonstrated by gene knockout experiments, which revealed significant changes in grain length and weight [30]. Additionally, *Vitis amurensis* (grapevine) studies have shown that the heterologous overexpression of *VaIAA3* significantly enhances cold tolerance [31].

In addition to developmental roles, *Aux*/*IAA* genes also play a crucial role in plant responses to abiotic stress. For instance, under salt stress, *OsIAA9*/*20* are induced, while *OsIAA7*/*8* are suppressed in rice [32]; in apple, *MdIAA3* and *MdIAA9* are up-regulated [33], whereas *GmIAA4* in soybean is down-regulated [34]. *AoIAA4* in *Asparagus* shows concentration-dependent suppression [35], while *AoIAA1*, *AoIAA10*, and *AoIAA12* are significantly upregulated under salt stress [36]. Similarly, drought stress upregulates *AtIAA30* in *Arabidopsis* [37], *OsIAA6*/*16* in rice [38], *CA03g04310* in pepper [39], *GmIAA57* in soybean [34], and *MsIAA3*/*5* in alfalfa [40], but represses most *SbIAA* genes in sorghum [41]. Notably, many members, such as *OsIAA1*/*9* in rice [38], *PmIAA14*/*17* in *Prunus mume* [41], *MtIAA14* in *Medicago truncatula* [42] and *VvAux*/*IAA4* in *Vitis vinifera* [2] respond coordinately to both salt and drought stresses, suggesting conserved regulatory mechanisms. Exogenous auxin also rapidly modulates their expression. For example, 24 *OsIAA* genes in rice [38], 86% of *BnIAA* genes in *Brassica napus* [43], *CA06g13860* in pepper [39], *PmIAA5*/*6*/*9*/*17*/*18* in *Prunus mume* [41], and 12 *AaIAA* genes in *Artemisia argyi* [44], show significantly up-regulated expression after IAA treatment. In sorghum roots, *SbIAA2*/*4*/*6*/*7*/*18* are highly up-regulated, while *SbIAA15* is down-regulated [45]. In summary, *Aux*/*IAA* genes respond to abiotic stresses and exogenous hormone treatments through a complex and dynamic regulatory network. An in-depth analysis of their molecular mechanism is important for the genetic improvement of crop stress resistance.

Although the functions of *Aux*/*IAA* genes in stress adaptation have been widely studied in many related solanaceous crops like tomato and pepper [39,46], their roles in eggplant remain largely unexplored. In this study, the first genome-wide analysis of *Aux*/*IAA* genes in the eggplant genome was conducted. Through integrated analyses of gene structure, conserved domains, chromosome distribution, and phylogenetics, the molecular evolutionary patterns of eggplant *Aux*/*IAA* genes were revealed. Furthermore, promoter *cis*-elements and multi-tissue expression profiles were analyzed. The biological functions of these members in stress responses were subsequently verified by analyzing their dynamic response patterns under drought, salt, and exogenous IAA treatments. The research results will provide a theoretical basis for elucidating the molecular mechanisms of stress adaptation and offer valuable candidate gene resources for stress-resistant molecular breeding in eggplant.

## 2. Results

### 2.1. Identification of SmIAA Genes in Eggplant Genome and Analysis of Protein Properties

A total of 40 candidate sequences were initially identified in the eggplant genome through BLASTP (version 2.15.0+) and HMM methods, using the Pfam database to confirm the presence of the Aux/IAA domain (PF02309). After removing sequences lacking the Aux/IAA domain using NCBI CDD, InterPro and SMART tools, 35 *Aux*/*IAA* family members were ultimately identified. These identified genes were systematically named SmIAA1 to SmIAA35 according to their chromosomal positions. Comprehensive physicochemical characterization revealed substantial variation among the family members (Table 1). Amino acid lengths ranged from 150 residues (SmIAA23) to 1108 residues (SmIAA25), with an average of 409.4. Molecular weights (MW) varied from 16.82 kDa (SmIAA23) to 122.6 kDa (SmIAA25). The theoretical isoelectric points (pI) spanned from 4.57 (SmIAA5) to 8.97 (SmIAA30), with 20 members classified as acidic proteins (pI < 7) and the remaining 15 members as alkaline proteins (pI > 7). The instability indices (II) ranged from 24.17 (SmIAA24) to 69.35 (SmIAA26), with the majority of proteins (28) predicted to be unstable (II > 40), and only seven members being stable proteins. The aliphatic index (AI) was between 62.26 (SmIAA33) and 91.73 (SmIAA5), indicating that all these members had good thermal stability. All members exhibited hydrophilic properties, as evidenced by GRAVY values ranging from −0.066 (SmIAA5) to −0.75 (SmIAA31). The in silico subcellular localization predictions indicated that SmIAA20 resides in the plasma membrane, SmIAA24 in the cytoplasm, and the remaining 33 members in the nucleus, suggesting diverse biological functions within the family.

### 2.2. Chromosomal Location and Phylogenetic Analysis of SmIAA Genes

**Figure 1:**
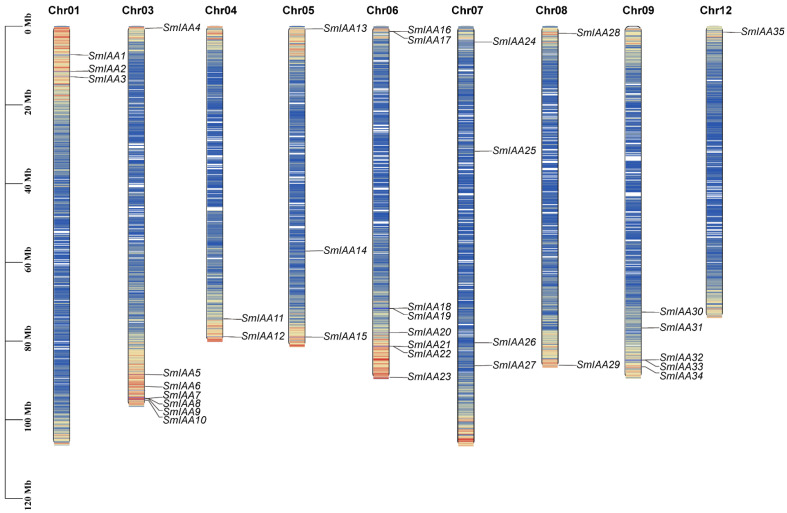
Chromosome location of the *SmIAA* genes.

Chromosomal mapping using TBtools-II revealed an uneven distribution of the 35 *SmIAA* genes across nine chromosomes, with none detected on chromosomes 2, 10, and 11 (Figure 1). Specifically, chromosome 6 harbored the highest number of *SmIAA* genes, with eight members, followed by chromosome 3 (seven genes), chromosome 9 (five genes), chromosome 7 (four genes), chromosomes 1 and 5 (three genes each), and chromosome 12 (only one gene). Most genes were clustered near chromosomal terminal regions, except *SmIAA14* and *SmIAA25*, which were localized to central regions. Notable gene clusters included *SmIAA7*/*8*/*9*/*10* on chromosome 3, *SmIAA16*/*17*, *SmIAA18*/*19*, and *SmIAA21*/*22* on chromosome 6, and *SmIAA32*/*33* on chromosome 9.

**Figure 2:**
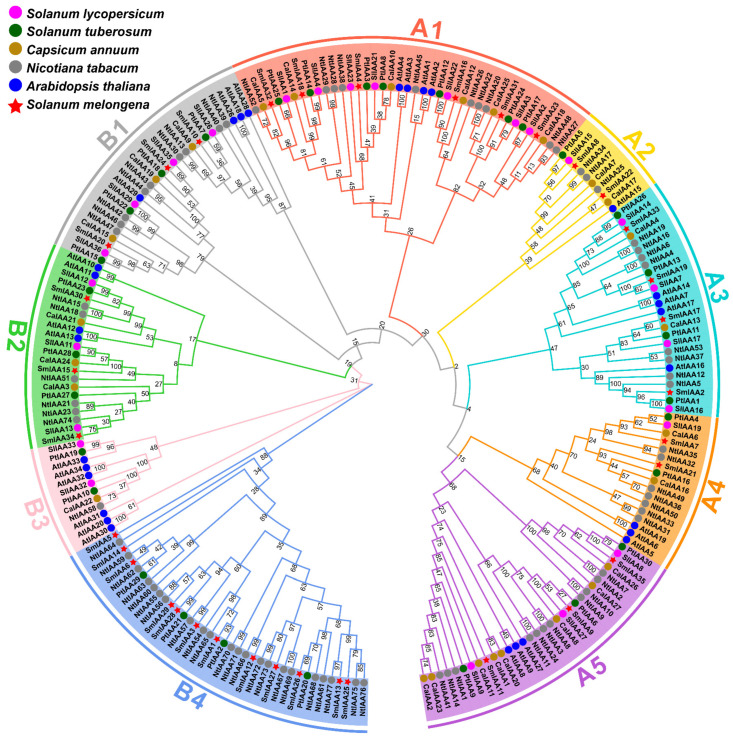
Phylogenetic tree of Aux/IAA proteins in six plant species. The evolutionary relationships of Aux/IAA proteins among *Arabidopsis* (blue circles), eggplant (red pentagrams), tobacco (gray circles), tomato (magenta circles), potato (dark green circles), and pepper (brown circles) are shown. Subgroups are marked with distinct colored backgrounds. The tree was constructed using the neighbor joining method in MEGA 11.0 with 1000 Bootstrap replicates.

To elucidate the evolutionary relationships of the *SmIAA* gene family in eggplant, a multiple sequence alignment of 223 Aux/IAA protein sequences from six species—(*Arabidopsis thaliana* 29), tomato (*Solanum lycopersicum*, 25), pepper (*Capsicum annuum*, 27), tobacco (*Nicotiana tabacum*, 77), potato (*Solanum tuberosum*, 30), and eggplant (*Solanum melongena*, 35)—was performed using MEGA 11.0. The optimal JTT model (Jones–Taylor–Thornton) was selected for constructing a Neighbor-Joining (NJ) phylogenetic tree (Figure 2). The tree divided proteins into two major clades (A and B), consistent with established *Arabidopsis* classifications. Clade A contained 124 proteins and was further subdivided into five subgroups (A1–A5), while Clade B (99 proteins) was separated into four subgroups (B1–B4). All subgroups in Clade A contained members from all six species, indicating a highly conserved core and a high degree of evolutionary conservation across Solanaceae plants. In Clade B, subgroups B1 and B2 included representatives from all six species, whereas B3 lacked eggplant members. Notably, subgroup B4 contained IAA proteins only from eggplant, potato and tobacco, suggesting lineage-specific diversification events in these three species.

### 2.3. Gene Duplication and Synteny Analysis of SmIAA Genes

**Figure 3:**
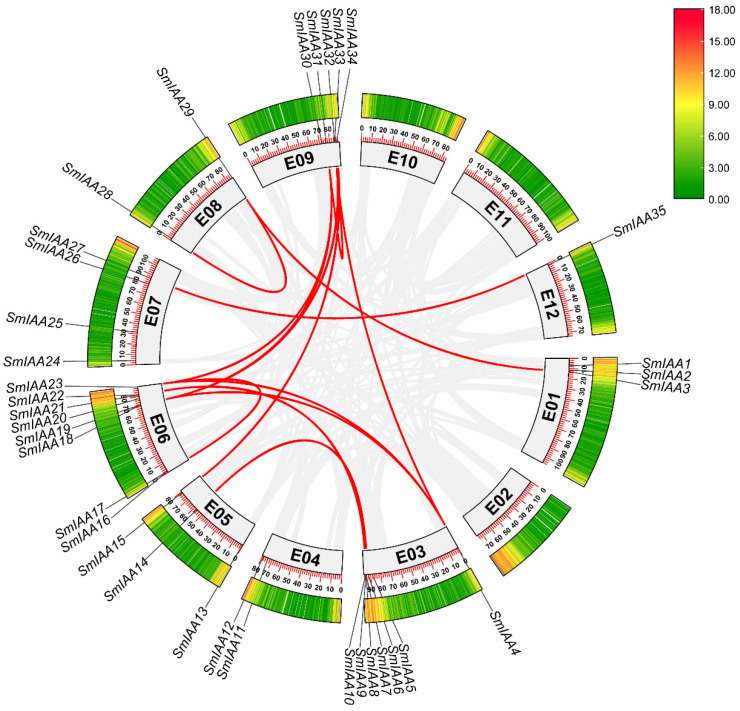
Synteny analysis of *SmIAA* genes in eggplant genome. The gray lines represent all the synteny blocks within the whole genomes. The red lines indicate collinear *SmIAA* gene pairs. The heat map represents gene density.

An intra-species synteny analysis was performed for the 35 eggplant *SmIAA* genes, which identified 13 segmental duplication events involving 15 *SmIAA* genes (Figure 3, Table S1). Three pairs occurred on the same chromosome, including *SmIAA16*/*SmIAA23* on chromosome 6, *SmIAA28*/*SmIAA29* on chromosome 8, and *SmIAA31*/*SmIAA32* on chromosome 9, while the remaining ten pairs resided on different chromosomes. Notably, eight duplication pairs belonged to subgroup A1, implicating segmental duplication as the primary expansion mechanism for this clade. To determine whether the *IAA* family genes were under the influence of selection pressure, the Ka/Ks (Ratio of nonsynonymous/synonymous) values of the *SmIAA* members was calculated. The results revealed Ka/Ks ratios < 1 for all 13 duplicated pairs, indicating predominant purifying selection. Despite observed chromosomal clustering, no tandem duplications were detected due to low sequence homology and structural divergence among clustered genes.

**Figure 4:**
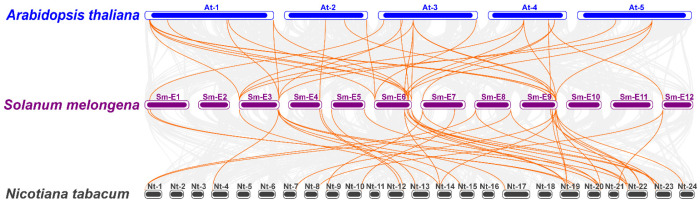
Collinearity analysis of *SmIAA* genes with *Arabidopsis thaliana* and *Nicotiana tabacum*. The gray background represents all synteny blocks within the whole genomes, and orange lines highlight the collinear gene pairs of *Aux*/*IAA* gene pairs.

To further clarify the evolutionary relationships among *IAA* genes in different species, inter-specific synteny analysis was performed for *IAA* genes in eggplant, *Arabidopsis* and tobacco (Figure 4, Table S2). The results revealed 39 syntenic gene pairs between eggplant and *Arabidopsis*, among which 20 *SmIAA* genes were collinear with 23 *AtIAA* genes. Subgroup A1 exhibited the highest collinearity, with 17 gene pairs. Eggplant and tobacco shared 42 syntenic pairs (26 *SmIAA* genes linked to 27 *NtIAA* genes), predominantly in subgroups A1 and B4 (10 pairs each). Notably, *SmIAA4* and *SmIAA32* showed syntenic relationships with multiple *AtIAAs* (*AtIAA2*/*3*/*4*/*14*), while *SmIAA32* additionally aligned with *NtIAA22*/*38*/*52*, demonstrating deep evolutionary conservation of *Aux*/*IAA* genes among different species.

### 2.4. Gene Structure and Conserved Motif Composition of SmIAA Genes

**Figure 5:**
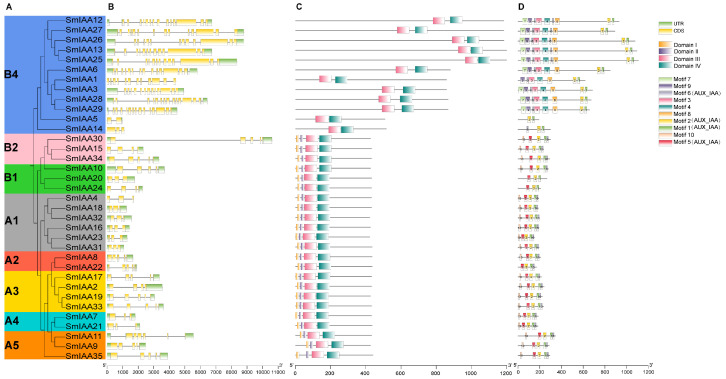
Evolutionary analysis, gene structure, conserved domains and motif structure of SmIAAs. (**A**) Phylogenetic tree of SmIAAs constructed using the NJ method with 1000 bootstrap replicates. (**B**) Gene structure of *SmIAA* genes. (**C**) Distribution of conserved domains. (**D**) Motif composition predicted by the MEME database; motifs 1–10 are indicated with different colors.

To characterize structural features of the *SmIAA* gene family, a phylogenetic tree of 35 SmIAA proteins was reconstructed using MEGA 11.0, confirming the absence of genes in subgroup B3 (Figure 5A). Gene structure, including the exon, intron and UTR composition of *SmIAAs* was diagrammed using TBtools-II (Figure 5B). Only three genes (*SmIAA1*/*4*/*14*) lacked annotated UTRs based on the current genome annotation. Exon numbers in the 35 *SmIAAs* varied significantly, ranging from 2 to 14. Subgroups A1 and A4 predominantly contained genes with three exons, while A2 and A3 mainly contained four exons, suggesting that genes within the same subgroup might have undergone similar evolutionary events. Notably, genes in subgroup B4 exhibited significantly higher exon counts, implying that they may encode proteins with more complex structures.

Analysis of Aux/IAA domains revealed that while typical Aux/IAA proteins contain four domains (I–IV), B4 proteins lacked domains I and II (Figure 5C and Figure S1), classifying them as non-canonical Aux/IAAs with potential specialized functions. To further characterize conserved sequence features within Aux/IAA domain regions, motif prediction analysis was performed using the MEME suite. MEME analysis identified ten conserved motifs (Figure 5D), with 35 SmIAA proteins harboring 2–9 motifs. SmIAA5 contained the fewest motifs (2), while five B4 members (SmIAA12/13/25/26/27) contained the maximum number of motifs (9). Motif annotation based on CDD and SMART databases indicated that Motifs 1, 2, 5, and 6 correspond to conserved Aux/IAA domain regions (Table S3). Motif 1 and Motif 2 were universally conserved across all members. Motif 6 was absent only in SmIAA5/20/24, whereas Motif 5 was specifically missing in B4 proteins. Motif 10 was absent from B4 members and SmIAA20/24. Motifs 3, 4, 7, 8, and 9 were exclusively present in B4 members. Conserved motif order within subgroups indicated the structural conservation during evolution in each subgroup.

### 2.5. Cis-Acting Element Analysis in Promoter Region of SmIAA

**Figure 6:**
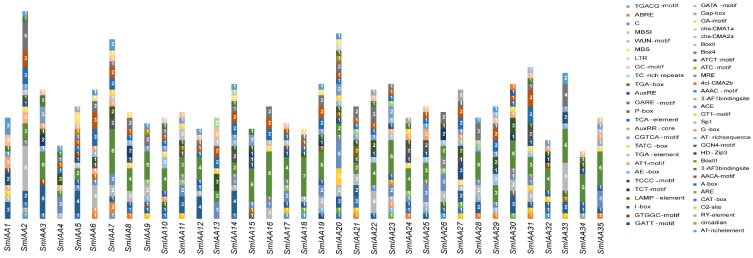
Distribution of *cis*-acting elements in SmIAAs promoters. *Cis*-acting elements were predicted within the 2000 bp promoter region upstream of each *SmIAA* gene. Elements were color-coded by type, and bar numbers represent element counts.

To investigate the potential transcriptional regulation mechanisms of *SmIAA* genes, 2 kb sequences upstream of the transcription start sites for all 35 *SmIAA* genes were extracted using TBtools-II. A total of 729 *cis*-acting elements representing 56 functional categories were identified (Figure 6 and Figure S2, Table S4). Light-responsive elements accounted for the highest proportion (54.0%), with Box4 element being the most frequent (133 occurrences across 35 genes). Hormone-responsive elements (23.9%) included auxin-responsive elements with gene-specific distribution: AuxRR-core exclusively present in *SmIAA21* and *SmIAA23*; AuxRE element only in *SmIAA13*, and TGA-box solely in *SmIAA10* and *SmIAA20*. Elements associated with cell development (15.0%) and stress responses (7.1%) were less abundant. Most genes (18/35) contained ≥20 elements, ranging from 37 (*SmIAA2*) to 12 (*SmIAA34*). Thirty-two genes harbored ≥10 distinct element types, with maximum diversity in *SmIAA20* (22 types) and minimum in *SmIAA34* (8 types). This highly heterogeneous distribution of *cis*-elements suggests that the members of this family may govern development and stress adaptation through divergent transcriptional regulation mechanisms.

### 2.6. Secondary and Tertiary Structure of SmIAA Protein

**Figure 7:**
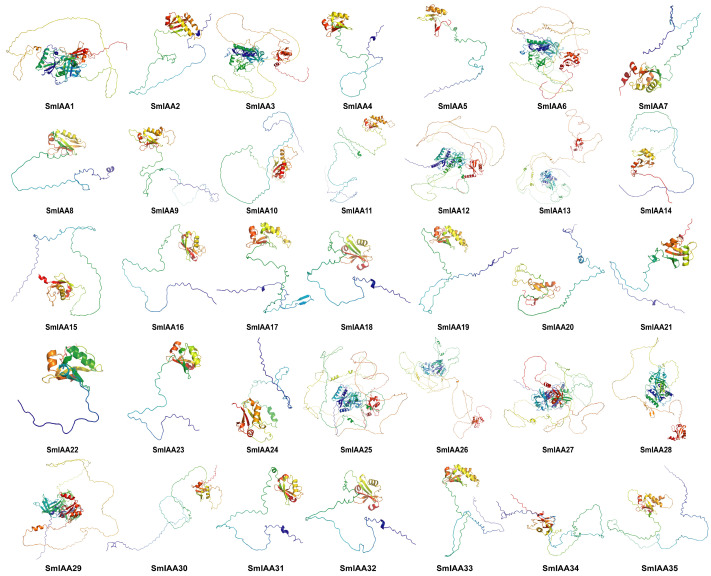
Three-dimensional structure diagrams of SmIAA proteins generated using PyMOL software (Version 3.1.6.1). Color scheme: α-helices (red), β-sheets (yellow), random coils (white), and β-turns (green).

A comprehensive structural characterization of SmIAA proteins was performed to elucidate potential functional mechanisms. Secondary structure prediction via SOPMA platform revealed significant compositional variation among family members (Table S5). Random coils were predominant across all proteins (58.29–80.81%), followed by α-helices (9.57–24.06%) and extended strands (8.23–22.67%). This prevalence of random coils implies that these proteins have a high degree of structural flexibility, potentially enabling multifunctional roles. Tertiary structures modeled in SWISS-MODEL and visualized in PyMOL confirmed extensive random coil regions, consistent with secondary structure predictions (Figure 7, Table S6). Pairwise RMSD analysis of 595 protein pairs identified 75 pairs with RMSD < 2 Å (Table S7). Among these, 50 pairs exhibited near-identical folds (RMSD < 1 Å), and 25 pairs showed high structural similarity with localized variations (RMSD 1–2 Å). Notably, 67 low-RMSD pairs (89.3%) occurred between different phylogenetic clades, with only eight intra-clade pairs (10.7%), seven of which were within subgroup A1. This distribution demonstrates that structural conservation extends beyond evolutionary clades, with limited correlation between structural similarity and phylogenetic proximity.

### 2.7. Protein–Protein Interaction Network Analysis

**Figure 8:**
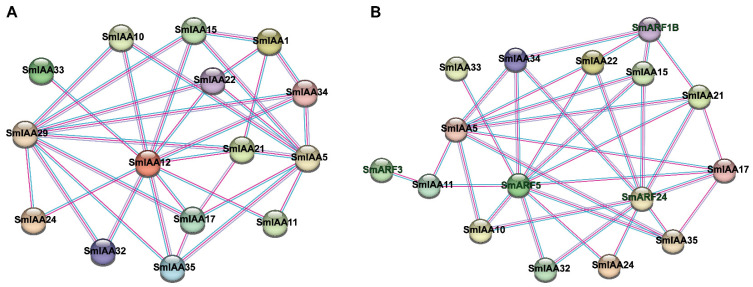
Protein–protein interaction networks in eggplant. (**A**) Interactions among SmIAA proteins. (**B**) Interactions between SmIAA and SmARF proteins. Nodes represent SmIAA (black) or SmARF (green) proteins. Edges indicate interactions supported by experimental evidence (pink) or predicted (blue).

In order to examine the potential interaction between SmIAA and SmARF proteins, a protein–protein interaction network for SmIAA-SmIAA and SmIAA-SmARF was predicted using STRING (Figure 8, Table S8). With a minimum interaction score of 0.7, a total of 34 SmIAA-SmIAA interaction pairs were identified. Among them, SmIAA12 emerged as a major network hub which interacted with 11 SmIAA proteins, followed by SmIAA29 with nine proteins, and SmIAA5 with eight proteins. For SmIAA-SmARF interactions, 35 interaction pairs were identified. Notably, four IAA proteins (SmIAA15, SmIAA21, SmIAA22, and SmIAA34), interacted with SmARF1B, SmARF24, and SmARF5 simultaneously, suggesting their involvement in core auxin-response networks. SmARF3 interacted exclusively with SmIAA11, implying that they may form a potentially independent and highly specific regulatory pathway.

### 2.8. Expression Patterns of SmIAA Genes

**Figure 9:**
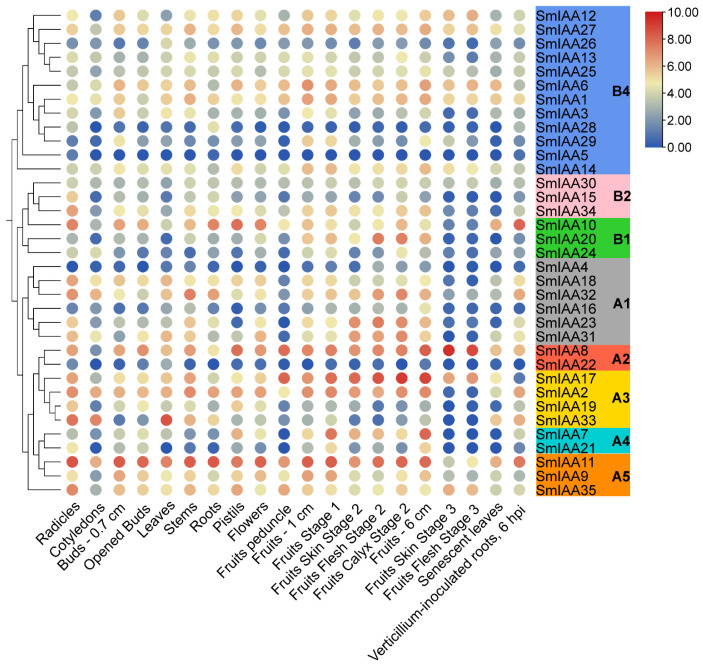
Expression heatmap of *SmIAA* genes across various eggplant tissues generated using TBtools-II. Expression levels are represented as the log_2_-transformed values, with red indicating higher expression and blue indicating lower expression.

Transcriptome data of 20 different tissues/organs of eggplant were downloaded from NCBI (https://www.ncbi.nlm.nih.gov/bioproject/328564, accessed on 25 July 2025, ID: PRJNA328564). The FPKM values of 35 *SmIAA* genes were extracted to obtain the expression levels of these genes (Figure 9, Table S9). *SmIAA4*, *SmIAA5*, *SmIAA22*, and *SmIAA28* showed low expression levels across most tissues, likely due to spatio-temporal specificity or detection limitations. Conversely, *SmIAA8* and *SmIAA11* exhibited constitutive high expression in most tissues, suggesting their fundamental regulatory roles. Most genes displayed low expression levels in cotyledons, fruit pedicels, senescent leaves, the third stage of fruit peel and the third stage of fruit pulp. Subgroups A3, A5, and B2 showed high expression levels in radicles. Notably, *SmIAA2* and *SmIAA17* were highly expressed during early fruit development when the fruits were at stage 1 or 2, suggesting their functional importance in fruit initiation. Additionally, *SmIAA2*, *SmIAA8*, *SmIAA10*, *SmIAA11*, *SmIAA32*, and *SmIAA33* were highly expressed in the roots 6 hpi with *Verticillium*, suggesting their possible roles in disease defense response.

### 2.9. Expression Analysis of SmIAA Genes in Response to Abiotic Stress and Auxin

To investigate *SmIAA* functions in abiotic stresses and plant hormone responses, candidate genes were initially selected based on their high expression levels in root transcriptome data and the functional representation of their phylogenetic subgroup. Based on the results, nine genes, including *SmIAA18* (A1), *SmIAA8* (A2), *SmIAA2*/*33* (A3), *SmIAA11*/*35* (A5), and *SmIAA1*/*12*/*27* (B4), were selected for subsequent qRT-PCR analysis to evaluate their dynamic expression profiles in roots under salt stress (200 mM NaCl), drought stress (20% PEG 6000), and exogenous IAA treatment (100 μM).

**Figure 10:**
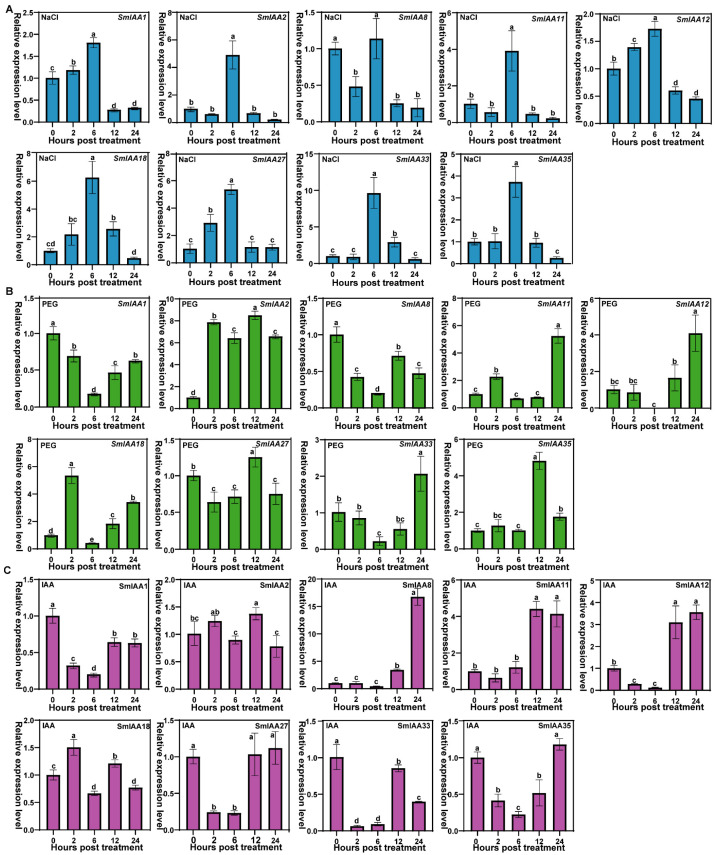
qRT-PCR analysis of *SmIAA* gene expressions under salt (**A**), drought (**B**), and IAA (**C**) treatments. Data are presented as the mean ± standard deviation (SD). Statistically significant differences (*p* < 0.05) among treatment groups were determined by Duncan’s test and are indicated by different lowercase letters.

Under salt-stress conditions (Figure 10A), all nine *SmIAA* genes exhibited differential responses, displaying three distinct temporal expression patterns. Most genes showed transient induction with a common peak at 6 h, suggesting that this time point is a critical phase of the salt-stress response. Among them, *SmIAA33* demonstrated the most significant induction (~8.6-fold), followed by *SmIAA18* (~5.2-fold), while *SmIAA8* initially exhibited suppression, followed by activation. A small subset, such as *SmIAA2*, displayed a delayed response with peak expression at 6 h. Under drought-stress conditions (Figure 10B), *SmIAA2*/*11*/*18* were significantly induced at the early stress stage (2 h), with *SmIAA2* showing sustained up-regulation (~7.8-fold), while *SmIAA1*/*8*/*27* were suppressed. By 6 h, most genes were downregulated and gradually recovered. Under IAA treatment (Figure 10C), *SmIAA1*/*27*/*33*/*35* were rapidly suppressed within the early stage (2 h), with *SmIAA33* decreasing 15.2-fold. Conversely, *SmIAA8*/*11*/*12* exhibited delayed up-regulation, peaking at 12–24 h. Notably, *SmIAA8* reached its peak at 24 h, with its expression level increasing by 15.7 times compared to the control. In contrast, *SmIAA2*/*18* displayed minimal expression changes. These divergent regulatory patterns demonstrate functional specialization of *SmIAA* genes in auxin signal transduction pathways.

## 3. Discussion

In this study, the first systematic identification and characterization of the *SmIAA* gene family in eggplant was conducted following the classification criteria established for *Arabidopsis IAA* genes [47]. Among the four Solanaceae species analyzed, eggplant harbors an intermediate number of *SmIAA* genes (35 members), exceeding those identified in tomato (25; [46]), pepper (27; [39]), and potato (30; identified in this study, Table S10), but significantly fewer than tobacco (77; [48]). This disparity likely stems from genome expansion driven by whole-genome duplication (WGD) events during the evolution of tobacco as an allotetraploid species. Although IAA gene families have been characterized in over 50 plant species, their phylogenetic classification systems show significant variation. The number of classified subgroups varies from 5 to 13, with diverse naming conventions (e.g., I–X, Clade A/B, Group 1–13 or A–J), lacking a unified standard. The *Arabidopsis* A/B classification scheme was widely adopted [16,42,48,49], in which Clade A mainly comprises structurally intact canonical Aux/IAA proteins involved in classical auxin signal transduction, whereas Clade B consists of more structurally diverse members that often lack key conserved domains [47]. Based on this framework, all 223 *IAA* genes from *Arabidopsis* and the five Solanaceae species were divided into two major clades: Clade A (subgroups A1–A5) and Clade B (subgroups B1–B4). Phylogenetic analysis reveals that Clade A, along with subgroups B1 and B2, encompasses IAA members from all five studied Solanaceae species and exhibited conserved domain architectures, suggesting evolutionary conservation of essential biological functions. Notably, significant divergence is observed among the other B subgroups: subgroup B3 lacks eggplant members, while subgroup B4 exclusively contains *IAA* genes from eggplant, tobacco, and potato. This phylogenetic distribution indicates distinct evolutionary events for subgroups B3 and B4 during Solanaceae evolution. Members of subgroup B4 are particularly striking due to their significantly higher gene structural complexity, characterized by the greatest number of exons and the most abundant motif composition, underscoring their unique evolutionary path.

The SmIAA proteins generally possess the four canonically conserved domains typical of Aux/IAA proteins [50]. However, consistent with their phylogenetic divergence, all 12 genes within subgroup B4 encode non-canonical Aux/IAA proteins lacking domain I and domain II, a structural feature that is likely associated with distinct regulatory properties. This finding aligns with studies of non-canonical proteins in *Arabidopsis*, where the absence of conserved domains disrupts classical auxin response mechanisms [51]. For example, *Arabidopsis* possesses six non-canonical *IAA* genes (classified here in subgroups B3/B4): AtIAA20/30/31/32/33/34. AtIAA20 (lacking domain II) exhibits resistance to rapid degradation under high auxin conditions [52]. AtIAA32/34 (lacking domain II) interacts with the receptor-like kinase TMK1 to participate in auxin signaling [53]. AtIAA33 (lacking domains I and II) maintains transcriptional repression of ARF10/16 by competitively repressing AtIAA5 [54]. Together, these findings illustrate that non-canonical Aux/IAA proteins can acquire novel regulatory functions through domain reorganization, thereby expanding the complexity of auxin signaling networks. In line with these observations, Phylogenetic analysis has identified multiple B4 clade members in potato, tobacco, and eggplant, suggesting a lineage-specific expansion of this subgroup within Solanaceae. Although functional evidence remains limited, available studies indicate that B4 members often exhibit regulatory mechanisms distinct from those of canonical Aux/IAA proteins. For instance, in potato, several B4 genes, including *PtIAA2* (*StPHYB*), *PtIAA20* (*StCDPK1*), *PtIAA21* (*StSUT4*), and *PtIAA29* (*StSP6A*), have been implicated in tuber development through roles in photoperiod perception, calcium signal transduction, sucrose transport, and tuber initiation, respectively [55]. In tobacco, NtIAA26 was shown to enhance salt tolerance by modulating potassium ion uptake and antioxidant activity [56]. By contrast, in eggplant, however, current knowledge is primarily limited to SmIAA1, which is upregulated during early fruit development and is predicted to encode SmARF1, suggesting a potential deviation from the canonical auxin signaling pathway [57]. Collectively, these observations suggest that certain B4 clade members may have evolved specialized regulatory functions. Nevertheless, the functional characterization of most B4 members remains to be further elucidated.

Analysis of *cis*-acting elements in the *SmIAA* promoter regions revealed their potential regulatory roles. A total of 56 distinct types of *cis*-acting elements were identified. Among these, numerous stress-responsive *cis*-elements were detected, including Low-temperature-responsive elements (LTR), drought-inducibility elements (MBS), wound-responsive elements (WUN-motif), anoxic-specific induction elements (GC-motif) and defense and stress-responsive elements (TC-rich repeats). These findings indicate that *SmIAA* genes likely play key roles in mediating plant responses to abiotic stresses during growth and development. Furthermore, plenty of *cis*-elements primarily associated with various plant hormone responses were identified, underscoring the complex relationship between the *SmIAA* gene family and hormonal regulation. Specifically, regarding auxin signaling, it was found that only one gene (*SmIAA13*) possessed the AuxRE element, critical for Aux/IAA binding to downstream ARFs in their promoters. Other auxin-responsive elements were identified in specific genes, including AuxRR-core elements in *SmIAA21*/*23*; TGA-box elements in *SmIAA2*/*7*/*11*/*16*/*17*/*35*, and a TGA-element in *SmIAA10*/*20*. These findings suggest that *SmIAA* genes not only play a role in auxin signaling but may also be involved in other phytohormone signal transduction [40,41].

Secondary structure analysis demonstrates that random coils predominate across all SmIAA proteins, accounting for 58.29–80.81%. Among the 35 SmIAA members, 22 proteins exhibit a composition pattern of “random coil > α-helix > extended strands”, which is consistent with the observations in alfalfa Aux/IAA proteins [40]. Tertiary structure comparisons using RMSD values revealed that only 50 out of 595 protein pairs showed high structural similarity (RMSD < 1 Å). Surprisingly, most of these pairs spanned different phylogenetic subgroups, with only seven pairs belonging to the same subgroup (A1). This finding contrasts with studies of other protein families, such as radish CKX [58], Alfalfa JAZ [59], Tobacco NF-Y [60], and Madhuca longifolia NAC [61], where intra-group structural conservation is typically observed. Although *SmIAA* genes within the same subgroup share similar gene structures and domain compositions, whether this structural heterogeneity is unique to eggplant or common among Aux/IAA proteins remains unclear and merits further study.

Protein–protein interaction (PPI) network analysis further revealed potential signaling modules. Substantial evidence confirms that Aux/IAA proteins typically regulate downstream target genes through heterodimerization with ARF proteins, mediated by Domains III and IV [62]. This regulatory mechanism is highly conserved across plant species and has been well documented in *Arabidopsis* as well as woody and horticultural plants, where Aux/IAA–ARF interactions participate in both developmental regulation and stress responses [16,63,64,65,66,67]. In this study, PPI predictions identify a core regulatory module comprising SmIAA21/22/15/34, which collectively interact with SmARF5/11/24, indicating that these genes play important roles in auxin signal transduction. Although there are no experimental reports on the interaction between IAA and ARF proteins in eggplant, the protein-interaction prediction in this study can provide a reference for subsequent experiments. Surprisingly, *SmIAA13* is the only gene containing the AuxRE element, which is a critical site for Aux/IAA-ARF interactions, while showing no predicted ARF binding. This discrepancy necessitates experimental validation to determine whether it reflects a unique regulatory mechanism in eggplant.

The *SmIAA* gene family exhibits sophisticated regulatory networks governing both tissue development and stress responses. Tissue-specific expression profiling revealed that *SmIAA2* and *SmIAA17* are significantly upregulated during early fruit development, suggesting their involvement in regulating cell division and fruit initiation. This observation aligns functionally with mechanisms reported for the kiwifruit (*Actinidia chinensis*) *AcIAA* family in modulating fruit maturation [68] and the stage-specific expression of *Dendrobium officinale DoIAA* genes during floral organogenesis [67], underscoring the conserved role of *Aux*/*IAA* genes in orchestrating organ development through spatiotemporal expression divergence.

Regarding abiotic stress responses, all the tested genes reached maximum induction levels at the 6 h post-salt treatment, suggesting a critical role for this time point. This pattern is consistent with previous studies on rice *OsIAA* genes under drought stress [38] and the 3–6 h gene activation cycle observed in apple roots under salt stress [69]. These findings suggest that early signaling pathways for abiotic stress are evolutionarily conserved across species. Conversely, the response of *SmIAA* genes to drought stress showed divergent trends at the early stage (2 h): a significant suppression of *SmIAA1*/*8*/*27*, which contrasted with the strong induction of *SmIAA2*/*11*/*18* (*SmIAA2* upregulated 7.8-fold). This diversity in expression has been conserved across rice [38], sorghum [45], and alfalfa [42] families, indicating that *Aux*/*IAA* genes likely employ dual regulatory mechanisms (induction and suppression) to modulate drought adaptation. Notably, the pronounced responsiveness of *SmIAA18*/*33* to salt and *SmIAA1*/*2*/*8* to drought highlights their potential as key candidate genes for deciphering abiotic stress tolerance mechanisms in eggplant. Exogenous IAA treatment induced a bidirectional expression pattern: *SmIAA1*/*27*/*33*/*35* were sharply downregulated within 2 h (*SmIAA33* was suppressed 15.2-fold), aligning with the canonical SCF^TIR1^-mediated ubiquitination degradation pathway [25,26]. In contrast, *SmIAA8*/*11*/*12* exhibited significant delayed induction. This differential responsiveness to exogenous auxin, consistent with observations across diverse species [43,44,45,70], demonstrates that the *Aux*/*IAA* family employs specialized regulatory strategies to finely coordinate growth and stress adaptation. The rapid suppression of *SmIAA33* and the marked delayed induction of *SmIAA8* are particularly noteworthy, suggesting their pivotal roles within the auxin signaling pathway. These prioritized candidates warrant an in-depth functional study to elucidate their mechanisms in eggplant development and stress resistance. Interestingly, *SmIAA1*/*12*/*27*, all belonging to subgroup B4, exhibited identical expression patterns, suggesting conserved functional roles for this subgroup in responding to abiotic stress and exogenous IAA treatment.

The roles of the *SmIAA* genes in auxin signaling and abiotic stress responses revealed in this study provide valuable candidate gene resources for molecular breeding in eggplant. Notably, genes such as *SmIAA18*/*33* and *SmIAA1*/*2*/*8*, which show significant responses to salt and drought stress, could be functionally validated through gene editing or transgenic technologies, enabling the development of eggplant varieties with enhanced stress tolerance. Moreover, screening for high-expression variants or stress-tolerant mutants of *SmIAA* genes could offer new strategies for improving the environmental adaptability of eggplant.

## 4. Materials and Methods

### 4.1. Genome-Wide Identification of SmIAA Gene Family in Eggplant Genome

The genome sequence, protein sequences and annotation files for eggplant (*Solanum melongena*, SME-HQ) were retrieved from the Eggplant Genome Database (http://eggplant-hq.cn/, accessed on 4 March 2024). *Arabidopsis thaliana* *Aux*/*IAA* gene and protein sequences were sourced from genes reported by Overvoorde et al. (2005) [19] and downloaded from the TAIR database (https://www.arabidopsis.org/, accessed on 4 March 2024). Sequences of other Solanaceae crops, including tomato (*Solanum lycopersicum*, ITAG2.4; [46]), pepper (*Capsicum annuum*, CAN_r1.2; [39]), tobacco (*Nicotiana tabacum*, Nitab-v4.5; [48]), and potato (*Solanum tuberosum*, DM v6.1; [71]) were obtained from the Sol Genomics Network (https://solgenomics.net/, accessed on 4 March 2024). The Hidden Markov Model (HMM) profile for the Aux/IAA domain (PF02309) was downloaded from the Pfam database (http://pfam.xfam.org/, accessed on 11 March 2024) [72]. Using 29 *Arabidopsis* Aux/IAA proteins as reference sequences, candidate genes were identified through BLASTP and HMM screening (E-value ≤ 1 × 10^−5^) performed using TBtools-II (Version 2.376) [73]. Sequences lacking the Aux/IAA domain were subsequently filtered out using the InterPro (https://www.ebi.ac.uk/interpro/, accessed on 11 March 2024), CDD (https://www.ncbi.nlm.nih.gov/cdd/, accessed on 11 March 2024), and SMART (https://smart.embl.de/, accessed on 11 March 2024) databases, resulting in the final set of *Aux*/*IAA* family members. Physicochemical properties of the identified SmIAA family members, including the number of amino acids (AA), molecular weight (MW), isoelectric point (pI), instability index (II), aliphatic index (AI), and grand average of hydrophobicity (GRAVY), were analyzed using TBtools-II. In silico subcellular localization predictions were performed using the online tool CELLO (http://cello.life.nctu.edu.tw/, accessed on 28 July 2024) [74].

### 4.2. Chromosomal Localization and Phylogenetic Analysis

Based on genomic annotation data of each *SmIAA* gene in eggplant, chromosomal distribution maps were generated using TBtools-II with a 2 kb sliding window for density analysis. To examine evolutionary relationships among Aux/IAA proteins from eggplant, *Arabidopsis*, tomato, pepper, tobacco, and potato, protein sequences were aligned using MEGA 11.0 [75]. Amino acid substitution model evaluation was performed in MEGA 11.0, and the JTT substitution model, which showed the best fit for the aligned protein sequences, was selected to construct a Neighbor-Joining (NJ) phylogenetic tree with 1000 bootstrap replicates. The resulting tree was visualized using EVOLVIEW (https://www.evolgenius.info/evolview/#/, accessed on 17 August 2024).

### 4.3. Gene Duplication and Synteny Analysis

Intra- and inter-species collinearity analyses were performed using the OneStep MCScanX-SuperFast module in TBtools-II with default parameters based on the Nei–Gojobori (NG) model with 1000 bootstrap replicates under default settings, based on the whole-genome sequences and annotation files of eggplant, *Arabidopsis*, and tobacco, to obtain segmentally and tandemly duplicated genes. The synonymous substitution rate (Ks) and nonsynonymous (Ka) substitution rate (Ka) for duplicated genes were subsequently calculated using the Simple Ka/Ks Calculator in TBtools-II based on the Nei–Gojobori (NG) model with 1000 bootstrap replicates under default settings, to estimate evolutionary rates and selective pressures acting on duplicated *SmIAA* genes.

### 4.4. Gene Structure and Conserved Motif Analysis

Based on genomic and protein sequences of *SmIAA*, TBtools-II was employed to visualize the gene structures of *SmIAA* genes, including the distribution of exons, introns, and untranslated regions (UTRs). Protein conserved motif analysis was performed using the online tool MEME (http://meme-suite.org/, accessed on 31 July 2024) [76,77], using the classic motif discovery mode, with the maximum number of motifs set to 10, while all other parameters were kept at default settings, with a maximum of 10 motifs and default settings for other parameters. Multiple sequence alignment was conducted using Clustal Omega (https://www.ebi.ac.uk/jdispatcher/msa/clustalo/, accessed on 1 August 2024). The resulting alignment files were subsequently imported into Jalview (Version 2.11.5.1) [78] for further analysis. In Jalview (Version 2.11.5.1), a default conservation threshold of 30% and the Clustal color scheme were applied to visualize residue conservation. Consensus sequences and sequence logos were generated using built-in functions to evaluate amino acid frequency patterns in conserved regions. These results were integrated with gene structure visualization from TBtools-II for comprehensive interpretation.

### 4.5. Promoter Cis-Regulatory Element Analysis

A 2000 bp upstream region from the ATG start codon of each *SmIAA* gene was extracted using TBtools-II (Gtf/Gff3 Sequences Extract) with the “Up Stream Bases” parameter set to 2000, while other parameters were kept at default values. The promoter sequences were analyzed using PlantCARE with default settings, and cis-acting elements with sequence similarity ≥70% were retained (https://bioinformatics.psb.ugent.be/webtools/plantcare/html/, accessed on 3 August 2024). Core promoter elements (e.g., TATA-box, CAAT-box) were excluded from subsequent analysis [79]. The remaining elements were functionally categorized, and their distributions were visualized as stacked bar charts using pivot tables in Excel.

### 4.6. Protein Secondary and Tertiary Structure Prediction

Secondary structures of SmIAA proteins were predicted using SOPMA (https://npsa-prabi.ibcp.fr/cgi-bin/npsa_automat.pl?page=/NPSA/npsa_sopma.html/, accessed on 26 July 2025) [80] to estimate proportions of α-helices, extended strands and random coils. Tertiary structures of all 35 proteins were modeled using SWISS-MODEL. Templates were selected based on the following criteria: sequence identity > 70%, coverage > 70%, and GMQE score > 0.4 [81]. Resulting PDB files were visualized in PyMOL (version 3.1.6.1, cartoon mode; chain-specific coloring). Pairwise root-mean-square deviation (RMSD) [82] values were calculated for all protein structures, using PyMOL’s ‘align’ command via command-line interface.

### 4.7. Protein–Protein Interaction (PPI) Network Construction

Protein sequences of 35 SmIAA proteins identified in this study and 20 SmARF proteins previously reported in eggplant [83] were extracted from the eggplant proteome database. PPI networks (SmIAA-SmIAA and SmIAA-ARF) were predicted using STRING platform (http://string-db.org/ accessed on 5 July 2025) [84] with *Arabidopsis thaliana* selected as the reference species to infer interaction relationships based on orthology. The interaction sources included experimental evidence, curated databases, co-expression, gene neighborhood, gene fusion, and co-occurrence. Only interactions with a confidence score ≥ 0.7 (high confidence) were retained for further analysis. The resulting interaction networks were visualized and analyzed to identify putative core regulatory modules within the auxin signaling pathway.

### 4.8. Expression Profiling of SmIAA Genes

Transcriptome data (Project ID: PRJNA328564) encompassing 20 different tissues or organs, including radicles, cotyledons, 0.7 cm buds, opened buds, leaves, stems, roots, pistils, flowers, fruit peduncle, 1 cm fruit, fruit stage 1, fruit skin stage 2, fruit flesh stage 2, fruit calyx stage 2, 6 cm fruit, fruit skin stage 3, fruit flesh stage 3, senescent leaves, and Verticillium-inoculated roots (6 hpi), were downloaded from NCBI (https://ncbi.nlm.nih.gov/bioproject/328564, accessed on 25 July 2025). FPKM values were log_2_-transformed from the raw data, and tissue-specific expression profiles of *SmIAA* genes were visualized through hierarchical clustering heatmaps generated using TBtools-II.

### 4.9. qRT-PCR Analysis Under Stress Treatments

Seeds of the eggplant cultivar ‘Baiyeqie’, kindly provided by the Institute of Vegetables and Flowers, Chinese Academy of Agricultural Sciences (accession number: V06B1246), were germinated and grown under controlled environmental conditions (28 °C/20 °C day/night temperatures, 70% relative humidity, 12,000 lux light intensity, 8 h light/16 h dark photoperiod). For stress treatments, root of 4- to 6-leaf-stage seedlings were carefully cleaned and immersed in 200 mM NaCl (salt stress), 20% PEG6000 (drought stress), or 100 μM IAA (auxin treatment) [85,86,87,88]. Root samples were harvested at 0, 2, 6, 12, and 24 h post-treatment [39,86,88,89,90], immediately frozen in liquid nitrogen and stored at −80 °C. Each treatment consisted of three seedlings, with the 0 h sample serving as the control. Three independent experiments were performed to generate biological triplicates.

Total RNA was extracted from root samples using the SteadyPure RNA Kit (Accurate Biotechnology, Hunan, China). cDNA was synthesized using the Evo M-MLV Kit (Accurate Biology). Gene-specific primers for the 35 SmIAA genes were designed based on the CDS sequence using Primer 5 and synthesized by Sangon Biotech (Shanghai, China) (Table S11). qRT-PCR was conducted on an ABI 7500 system using SYBR Green Pro Taq HS Premix (Rox Plus, Accurate Biotechnology, Hunan, China) with the following protocol: 95 °C for 30 s, 40 cycles of 95 °C for 5 s, and 60 °C for 30 s. The *GAPDH* gene served as the internal control gene [85]. All qRT-PCR assays were performed in three independent biological and technical replicates. Relative expression level of each gene was quantified using the 2^−ΔΔCt^ method [91]. GraphPad Prism 8.0.2 software was employed to generate bar graphs and visualize the results.

## 5. Conclusions

In conclusion, this study systematically identified 35 *SmIAA* genes from the eggplant genome and classified them into two major clades (Clade A: subgroups A1–A5; Clade B: subgroups B1–B4) through comprehensive phylogenetic analysis. Detailed characterization encompassed their chromosomal distribution, gene structures, conserved domains, promoter *cis*-elements, collinearity relationships, and duplication events. Expression profiling across 20 eggplant tissues revealed distinct spatiotemporal patterns: four genes (*SmIAA4*/*5*/*22*/*28*) exhibited constitutive low expression, while two genes (*SmIAA8*/*11*) showed constitutive high expression, and others showed strong tissue- specificity, notably *SmIAA2* and *SmIAA17* during early fruit development. Furthermore, qRT-PCR analysis under abiotic stresses and IAA treatment demonstrated that *SmIAA* genes deploy specialized response strategies. *SmIAA18*/*33* were strongly induced by salt stress, *SmIAA1*/*2*/*8* showed pronounced responsiveness to drought, while *SmIAA8* (delayed induction) and *SmIAA33* (rapid degradation) displayed contrasting regulation under auxin treatment. These genes represent prime candidates for functional validation in their respective signaling pathways. Collectively, our findings provide crucial insights into the molecular basis of SmIAA-mediated regulation in eggplant growth and stress adaptation, establishing a foundation for enhancing stress resilience through molecular breeding approaches.

## Figures and Tables

**Table 1:** The characteristics of the eggplant *Aux*/*IAA* gene family.

Gene Name	Gene ID	Chr.	Subgroup	AA (bp)	MW (Da.)	pI	II	AI	GRAVY	SL
SmIAA1	Smechr0100790.1	1	B4	617	69,106.35	5.99	61.34	71.07	−0.496	N
SmIAA2	Smechr0101209.1	1	A3	239	26,663.29	5.72	32.54	71.72	−0.436	N
SmIAA3	Smechr0101326.1	1	B4	685	76,563.58	5.9	51.94	77.66	−0.461	N
SmIAA4	Smechr0300045.1	3	A1	190	21,784.92	5.41	56.91	69.84	−0.647	N
SmIAA5	Smechr0302810.1	3	B4	191	21,446.3	4.57	52.57	91.73	−0.066	N
SmIAA6	Smechr0303268.1	3	B4	848	94,291.76	6.51	55.48	65.18	−0.636	N
SmIAA7	Smechr0303465.1	3	A4	185	20,977.98	7.59	49.36	72.11	−0.591	N
SmIAA8	Smechr0303466.1	3	A2	208	23,109.32	7.58	50.77	71.2	−0.582	N
SmIAA9	Smechr0303478.1	3	A5	287	31,540.62	6.47	42.06	74.74	−0.522	N
SmIAA10	Smechr0303536.1	3	B1	279	30,860.66	8.79	40.81	67.46	−0.727	N
SmIAA11	Smechr0402028.1	4	A5	345	36,957.8	8.51	46	68.46	−0.458	N
SmIAA12	Smechr0402457.1	4	B4	930	102,636.05	5.18	50.73	73.89	−0.427	N
SmIAA13	Smechr0500039.1	5	B4	1094	120,901.13	5.91	57.96	75.51	−0.57	N
SmIAA14	Smechr0501636.1	5	B4	297	33,271.28	5.22	50.27	64.65	−0.728	N
SmIAA15	Smechr0502523.1	5	B2	233	25,998.13	5.25	31.62	64.81	−0.609	N
SmIAA16	Smechr0600104.1	6	A1	191	21,692.69	7.61	44.04	75.97	−0.603	N
SmIAA17	Smechr0600105.1	6	A3	214	24,232.88	8.7	39.99	76.92	−0.519	N
SmIAA18	Smechr0601560.1	6	A1	187	20,923.9	5.5	53.68	75.08	−0.591	N
SmIAA19	Smechr0601563.1	6	A3	223	25,344.13	8.78	40.53	67.71	−0.635	N
SmIAA20	Smechr0601905.1	6	B1	265	31,162.69	5.91	37.96	88.94	−0.396	PM
SmIAA21	Smechr0602219.1	6	A4	182	21,008.87	8.58	51.09	69.51	−0.663	N
SmIAA22	Smechr0602220.1	6	A2	169	18,898.94	7.66	36.84	90.47	−0.117	N
SmIAA23	Smechr0603146.1	6	A1	150	16,821.25	6.58	40.09	72.8	−0.469	N
SmIAA24	Smechr0700239.1	7	B1	207	23,101.43	7.62	24.17	88.36	−0.435	C
SmIAA25	Smechr0700752.1	7	B4	1108	122,631.44	6.23	61.66	71.1	−0.574	N
SmIAA26	Smechr0701387.1	7	B4	1077	120,390.25	6.19	69.35	69.55	−0.666	N
SmIAA27	Smechr0701526.1	7	B4	891	98,726.06	6.13	60.62	74.74	−0.433	N
SmIAA28	Smechr0800141.1	8	B4	673	75,524.05	5.96	60.78	70.82	−0.538	N
SmIAA29	Smechr0802512.1	8	B4	658	73,713.25	6.41	47.95	71.23	−0.532	N
SmIAA30	Smechr0901634.1	9	B2	298	32,132.18	8.97	45.31	72.28	−0.403	N
SmIAA31	Smechr0901825.1	9	A1	193	21,830.65	7.62	60.27	69.69	−0.75	N
SmIAA32	Smechr0902295.1	9	A1	198	22,292.42	6.01	56.72	69.95	−0.675	N
SmIAA33	Smechr0902296.1	9	A3	235	26,208.04	8.11	40.85	62.26	−0.595	N
SmIAA34	Smechr0902429.1	9	B2	290	31,324.28	8.32	27.97	62.52	−0.703	N
SmIAA35	Smechr1200111.1	12	A5	291	31,908.28	8.3	45.8	66.98	−0.538	N

Note: AA, amino acids; MW, molecular weight; pI, isoelectric point; II, instability indices; AI, aliphatic index; GRAVY, grand average of hydropathicity; SL, in silico subcellular localization; N, nucleus; PM, plasma membrane; C, cytoplasm.

## Data Availability

All data generated or analyzed during this study are included in this article and Supplementary Materials. The genome sequences of *Solanum melongena*, *Solanum lycopersicum*, *Capsicum annuum*, *Nicotiana tabacum*, and *Solanum tuberosum* were downloaded from Sol Genomics Network (https://solgenomics.net, accessed on 4 March 2024). The protein sequences of *Arabidopsis thaliana* were downloaded from TAIR databases (https://www.arabidopsis.org/, accessed on 4 March 2024). The transcriptome data were obtained from NCBI (https://ncbi.nlm.nih.gov/bioproject/328564, accessed on 25 July 2025, ID: PRJNA328564).

## Supplementary Materials

The following supporting information can be downloaded at: https://www.mdpi.com/article/10.3390/ijms27010350/s1.

## Abbreviations

The following abbreviations are used in this manuscript:

AA	amino acids
AI	aliphatic index
ARF	Auxin Response Factor
AuxREs	auxin-responsive elements
BLASTP	Basic Local Alignment Search Tool for Proteins
CDS	Coding Sequence
cDNA	Complementary DNA
DBDs	DNA-binding domains
FPKM	Fragments Per Kilobase of transcript sequence per millions base pairs sequenced
GMQE	Global Model Quality Estimation
GRAVY	grand average of hydrophobicity
HMM	Hidden Markov Model
IAA	Indole-3-acetic acid
II	instability index
JTT	Jones–Taylor–Thornton
Ka	nonsynonymous substitution rate
Ka/Ks	Ratio of nonsynonymous/synonymous
Ks	synonymous substitution rate
MW	molecular weight
NJ	Neighbor-Joining
pI	isoelectric point
PPI	Protein–protein interaction
qRT-PCR	Quantitative real-time PCR
RMSD	Root means square deviation
SmIAA	*Aux*/*IAA* genes of *Solanum melongena*
TIR1/AFB	Transport Inhibitor Response 1/Auxin-Signaling F-box proteins
