# Supplementary figures and images for: Genome-Wide Identification and Expression Profiling of the Aux/IAA Gene Family in Eggplant (Solanum melongena L.) Reveals Its Roles in Abiotic Stress and Auxin Responses
Source: Int J Mol Sci. 2025 Dec 29;27(1):350. doi: 10.3390/ijms27010350 (PMC12786059; doi:10.3390/ijms27010350)

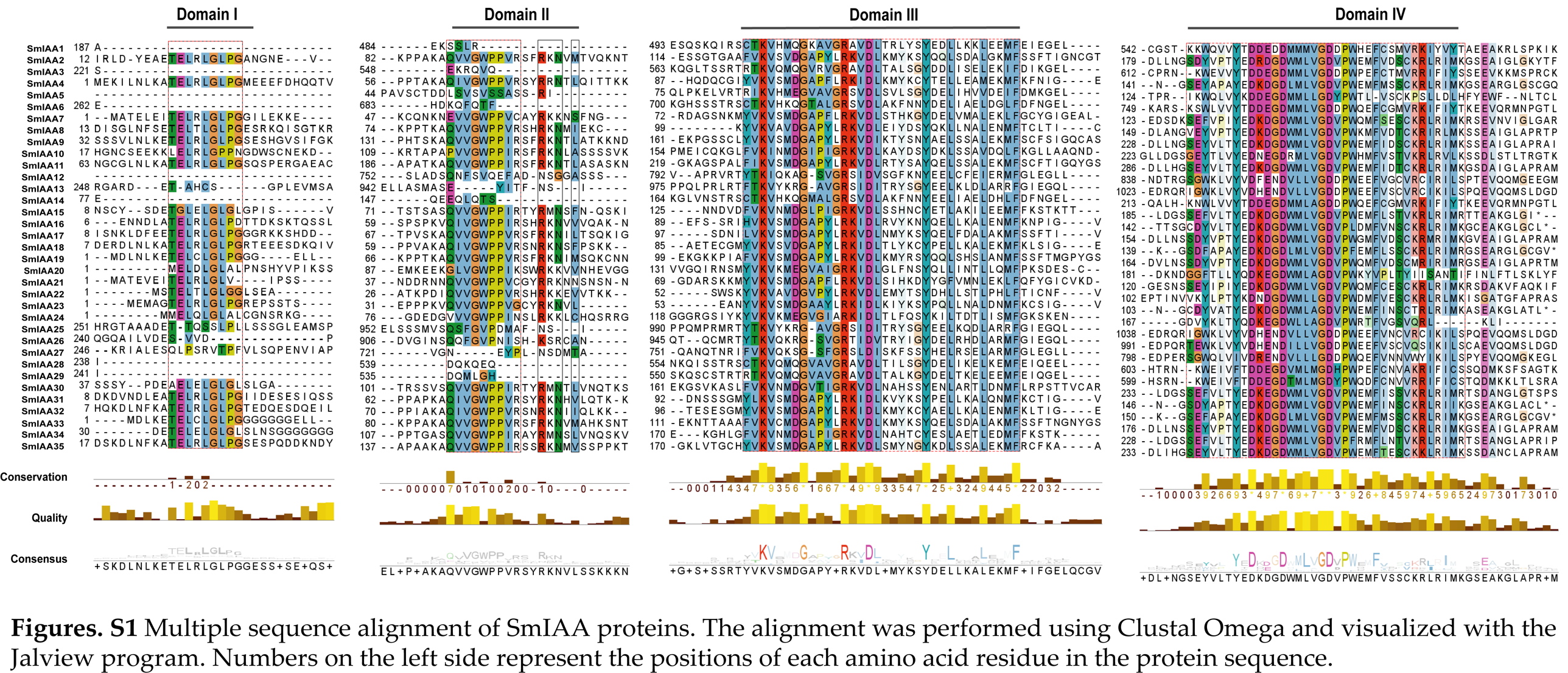

Supplement: Supplementary file 1 [file ijms-27-00350-s001.zip › Figures. S1 Multiple sequence alignment of SmIAA proteins.png]

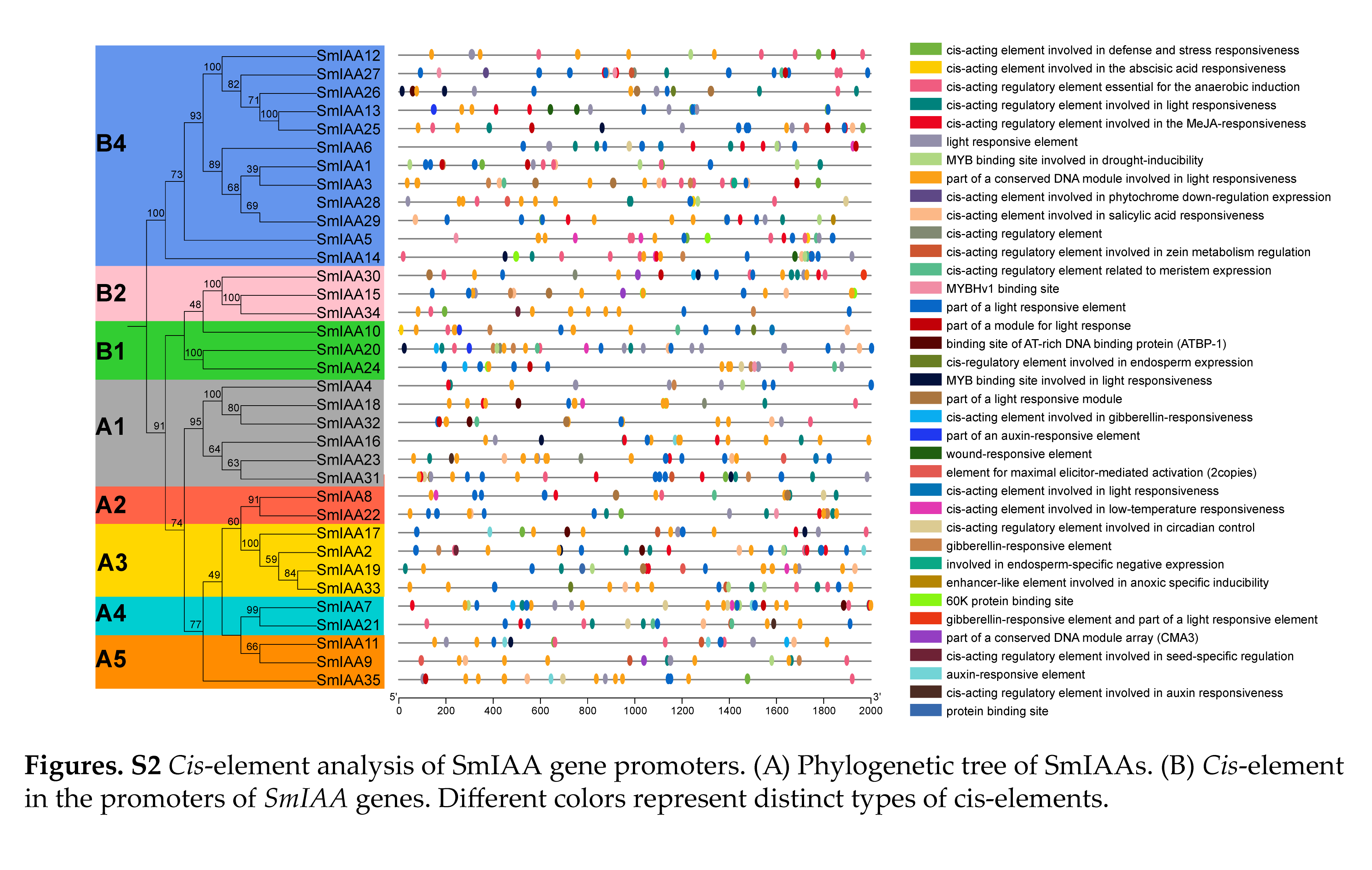

Supplement: Supplementary file 1 [file ijms-27-00350-s001.zip › Figures. S2 Cis-element analysis of SmIAA gene promoters.tif]
